# The Utilization of Heart Rate Variability for Autonomic Nervous System Assessment in Healthy Pregnant Women: Systematic Review

**DOI:** 10.2196/36791

**Published:** 2022-11-17

**Authors:** Zahra Sharifiheris, Amir Rahmani, Joseph Onwuka, Miriam Bender

**Affiliations:** 1 University of California, Irvine Irvine, CA United States

**Keywords:** heart rate variability, pregnancy, systematic review, autonomic nervous system assessment

## Abstract

**Background:**

The autonomic nervous system (ANS) plays a central role in pregnancy-induced adaptations, and failure in the required adaptations is associated with adverse neonatal and maternal outcomes. Mapping maternal ANS function in healthy pregnancy may help to understand ANS function.

**Objective:**

This study aimed to systematically review studies on the use of heart rate variability (HRV) monitoring to measure ANS function during pregnancy and determine whether specific HRV patterns representing normal ANS function have been identified during pregnancy.

**Methods:**

The Preferred Reporting Items for Systematic Reviews and Meta-Analyses (PRISMA) guideline was used to guide the systematic review. The CINAHL, PubMed, SCOPUS, and Web of Science databases were searched to comprehensively identify articles without a time span limitation. Studies were included if they assessed HRV in healthy pregnant individuals at least once during pregnancy or labor, with or without a comparison group (eg, complicated pregnancy). Quality assessment of the included literature was performed using the National Heart, Lung, and Blood Institute (NHLBI) tool. A narrative synthesis approach was used for data extraction and analysis, as the articles were heterogenous in scope, approaches, methods, and variables assessed, which precluded traditional meta-analysis approaches being used.

**Results:**

After full screening, 8 studies met the inclusion criteria. In 88% (7/8) of the studies, HRV was measured using electrocardiogram and operationalized in 3 different ways: linear frequency domain (FD), linear time domain (TD), and nonlinear methods. FD was measured in all (8/8), TD in 75% (6/8), and nonlinear methods in 25% (2/8) of the studies. The assessment duration varied from 5 minutes to 24 hours. TD indexes and most of the FD indexes decreased from the first to the third trimesters in the majority (5/7, 71%) of the studies. Of the FD indexes, low frequency (LF [nu]) and the LF/high frequency (HF) ratio showed an ascending trend from early to late pregnancy, indicating an increase in sympathetic activity toward the end of the pregnancy.

**Conclusions:**

We identified 3 HRV operationalization methods along with potentially indicative HRV patterns. However, we found no justification for the selection of measurement tools, measurement time frames, and operationalization methods, which threaten the generalizability and reliability of pattern findings. More research is needed to determine the criteria and methods for determining HRV patterns corresponding to ANS functioning in healthy pregnant persons.

## Introduction

The autonomic nervous system (ANS) is one of the central regulatory systems that responds to various internal and external stresses [1]^.^ Pregnancy is one of the stimuli that requires various physiological changes in order to adapt to relevant demands regarding fetal development and thus needs ANS regulatory function [2,3]. Systemic vasodilation is the primary initial pregnancy-related event [4-6]. The outcome of systemic vasodilation is a series of systemic accommodations that involve almost all the body systems including respiratory, cardiovascular, digestion, and endocrine systems [7]. The combination of these systemic accommodations is known as “homeostasis,” which is a dynamic and complex function [8]. Establishing homeostasis during pregnancy is necessary to promote embryotic and fetal growth. Due to the dynamic nature of pregnancy, it is critical to understand whether the corresponding dynamic ANS function results in a certain pattern of changes reflecting healthy accommodation in ways that can be observed and acted upon. However, the safeguard for the amount, severity, and pattern of safe changes in ANS function is not well known, in part because it is impractical to continuously observe the function in vivo using existing methods.

The traditional tests for ANS assessment are those that evaluate the cardiovascular reflexes in response to provocative maneuvers [9,10]. Although these ANS assessment maneuvers are widely applied in clinical settings for diagnostic purposes, the ability of these maneuvers to reflect ANS function in real life is not well-justified due to the fact that the tests are often performed in controlled situations and artificial settings such as laboratories and hospitals that intrinsically can affect ANS function during the assessment. Additionally, due to the dynamic nature of pregnancy-related accommodations, episodic-only assessments are insufficient to capture the dynamism in ANS function during pregnancy. Thus, to assess this dynamism, more in vivo assessment techniques in real time are needed.

Heart rate variability (HRV), defined as a variation in the beat-to-beat (RR or NN) interval, is a well-known, noninvasive assessment tool for ANS that has been recently applied widely for both clinical and nonclinical purposes [11]. A study performed with 8 million individuals indicated that HRV can vary by age, sex, and activity [12]. However, less is known regarding how pregnancy may affect HRV, to understand ANS regulations induced in pregnancy. This is especially important to investigate as various studies have indicated that ANS dysregulation assessed through HRV can be associated with common pregnancy complications including hypertensive disorders and gestational diabetes [13-16]. Thus, we aimed to review studies that have assessed HRV for ANS regulations during noncomplicated pregnancies to answer the following questions: (1) whether and how HRV has been used to measure ANS function during heathy pregnancy and (2) whether any specific HRV patterns have been identified during pregnancy.

## Methods

### Design

We conducted a systematic review using the Preferred Reporting Items for Systematic Reviews and Meta-Analyses (PRISMA) standards [17] to guide the study. The population, exposure, comparator, and outcome (PECO) framework was used to develop the research question and search terms. The research question was: Has HRV monitoring (exposure) been used to measure ANS function (outcome) during pregnancy (population), and if so, what specific HRV patterns (comparison) representing normal ANS function have been identified during pregnancy?

### Information Sources

The PubMed, CINAHL, SCOPUS, and Web of Science databases were searched initially in August 2020 and updated in June 2021 (past June 2021). Although we applied no limitation to the time span for the search, the time span varied for each database depending on the publication history of each database. See Multimedia Appendix 1 for more details.

### Search Strategy

To access further studies, additional sources were reviewed, including reference lists of the included articles and Google Scholar. Keywords including “Heart Rate Variability (HRV)” and “Pregnancy” were used for both simple and advanced searches of each database separately (see Multimedia Appendix 1 for all terms and search strategies used for each database). 

### Inclusion and Exclusion Criteria

The population included healthy pregnant individuals.

Studies that involved various interventions (eg, exercise) with healthy pregnant women were excluded. Being pregnant was considered as the exposure (E) component, which was required for all the studies. Studies with or without a comparison (C) group (eg, complicated pregnancy) were eligible for inclusion. HRV, assessed at least once during pregnancy or labor, was considered the expected outcome (O) for all the studies that were assessed. Studies were included if available in the English language. Exclusion criteria were systematic reviews, protocols, conference proceedings, letters to the editor, unpublished or under review papers, and dissertation proposals.

### Selection and Data Collection Process

Selected articles were peer reviewed in Covidence online software by 2 independent reviewers. To assess the relevancy, all the studies were screened by both reviewers, ZS and JO, based on titles, abstracts, and full text in 2 steps. In the first step, the abstracts of all the articles that were gathered from the databases were screened in terms of their relevance to our study aim. Next, those articles with relevant titles or abstracts from the first step underwent a full-text assessment. To resolve disagreements, a third reviewer, MB, was involved.

### Data Items

We collected the following data: HRV results, as the main outcome; assessment tools used to measure HRV; HRV component(s); frequency and duration of the assessment; and gestational age at the assessment.

### Effect Measures

The effect measure for all the studies was the mean difference, and a significant difference was considered at a *P* value <.05.

### Risk of Bias Assessment

Two independent reviewers, ZS and JO, assessed the methodological quality of the selected studies using the National Heart, Lung, and Blood Institute (NHLBI) Quality Assessment scale for observational cohort and cross-sectional studies [18]. The NHLBI quality assessment tool, consisting of 14 questions, assesses studies in terms of the following criteria: study objectives, study population, sample size, exposure, outcome measures, and key potential confounding variables. Each study was assessed for a risk of bias using responses of “yes,” “no,” and “cannot determine/not applicable/not reported” for every single criterion. 

### Synthesis Methods

The included studies were not homogenous in terms of the assessment time frame, component, and frequency; thus, a meta-analysis was not possible. A narrative synthesis was chosen to bring together the broad knowledge from a variety of approaches. This type of synthesis is not the same as a narrative description that accompanies many reviews. To synthesize the literature, we applied the guideline from Popay et al [19]. The steps include (1) preliminary analysis, (2) exploration of relationships, and (3) assessment of the robustness of the synthesis. Theory development was not performed due to the exploratory nature of the research synthesized. For the main synthesis, we extracted the descriptive characteristics of the included studies, presented them in a table, and produced a textual summary of the results. These characteristics included first author, publication year, country, study design, population, and sample size. Then, we applied thematic analysis to extract the main themes from all studies. The 3 themes presented in the Results section represent the main areas of knowledge available about HRV in pregnant individuals. These included HRV-related measures during pregnancy (duration and frequency of the HRV assessment, assessment tool used to measure HRV, and assessed HRV components), HRV changes or patterns in the different trimesters of pregnancy as compared with nonpregnant individuals, and HRV changes or patterns across or between the different trimesters of pregnancy.

## Results

### Study Selection

**Figure 1:**
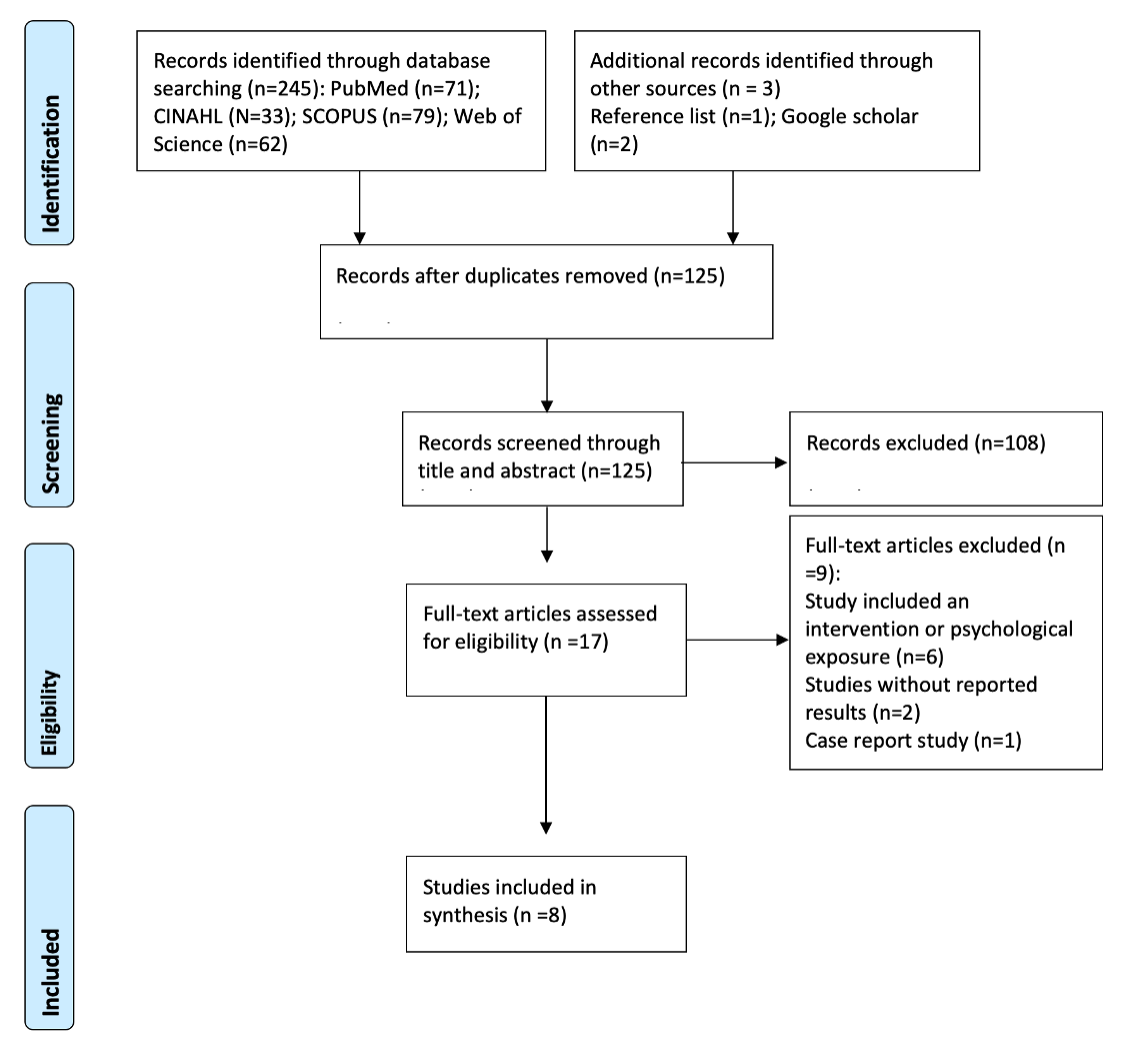
Preferred Reporting Items for Systematic Reviews and Meta-Analyses (PRISMA) flow chart for study selection.

A total of 245 articles were accessed, of which 120 duplicates were removed by 2 autonomous reviewers. Of the remaining 125 articles, 108 were excluded during the title and abstract screening process. Of the 17 articles that underwent the full-text screening process, 9 were excluded for a variety of reasons (intervention, psychological exposure, findings not reported), resulting in 8 articles that went through the data extraction and synthesis process: See Figure 1 for the PRISMA flow.

### Results of the Synthesis

#### Study Characteristics

Participants were mainly pregnant (as the study group) and sometimes included nonpregnant individuals (as a control group) aged from 16 years to 45 years. One of the studies included hypertensive and pre-eclamptic comparison groups [20]. All 8 studies included healthy participants as the main study group. The definition of healthy pregnancy varied from study to study; studies often were selective in considering the American College of Obstetricians and Gynecologists definition or did not specify the characteristics for their definition. For example, one of the studies [21] relied on 2 criteria for distinguishing healthy pregnant individuals: no history of cardiovascular diseases and no drug consumption affecting the ANS. All the studies recruited participants from outpatient settings including prenatal care centers. Of the 8 included studies, 5 were conducted in India, 2 were conducted in the United States, and 1 was conducted in Portugal. See Table 1 for details.

**Table 1:** Characteristics of included studies based on the population, exposure, comparator, outcome, and study (PECOS) framework.

Author, publication year, country	Study design (S, C)	Population (P, E)	Duration and frequency	Tool	HRV components (O)
Chamchad et al [22], 2007, United States	Cross-sectional with 2 comparison groups	24 nonpregnant healthy and 22 full-term (labor) healthy individuals with a single gestation	A single 10-minute supine position	ECG^a^	LF^b^ (ms^2),^ HF^c^ (ms^2^), LF/HF, mean NN interval, SDNN^d^, RMSSD^e^, pNN50^f^, HTI^g^, TINN^h^
Puente [20], 2011, Portugal	Longitudinal observational study with 3 groups	217 participants: 135 normal blood pressure, 55 hypertensive, 27 pre-eclamptic	563 recordings of 10-minute measurements at ≤14, 15-19, 20-24, 26-30, 30-35, 36-40 weeks of gestational age in the sitting position	ECG	LF (ms^2^), HF (ms^2^), LF/HF, VLF^i^
Garg et al [23], 2020, India	Longitudinal observational with a single group	66 healthy pregnant individuals	A 5-minute measurement at 11-13, 20-22, and 30-32 weeks of gestation in the supine position	ECG	LF (ms^2^), HF (ms^2^), LF (nu), HF (nu), LF/HF, SDNN, SDSD^j^, pNN50, total power (TP)
Alam et al [24], 2018, India	Cross-sectional with 4 groups	200 healthy participants: nonpregnant (n=50), first trimester (n=50), second trimester (n=50), and third trimester (n=50)	A single 5-minute measurement between 9:00 AM and 12.00 PM in the supine position	ECG	LF (ms^2^), HF (ms^2^), HF (nu), LF (nu) LF/HF, mean RR interval, SDNN, RMSSD, NN50, pNN50
Gandhi et al [25], 2014, India	Longitudinal observational with 2 comparison groups	60 healthy participants: pregnant individuals (n=30), nonpregnant individuals (n=30)	A single 5-minute measurement for nonpregnant individuals and twice in the first (6-12 weeks) and third (25-36 weeks) trimesters for pregnant individuals	ECG	VLF, LF (ms^2^), HF (ms^2^), LF (nu), HF (nu), LF/HF, SDNN, RMSSD, SDSD, NN50, pNN50, SD_1_^k^/SD_2_^l^, HTI, mean RR interval
Solanki et al [21], 2020, India	Cross‑sectional case-control study	119 healthy individuals: pregnant individuals (n=89): T_1_ (n=24), T_2_ (n=37), T_3_ (n=28), and nonpregnant individuals (n=30)	A single 5-minute assessment between 8.30 am and 12.00 pm in the supine position	ECG	VLF, LF (nu), HF (nu), LF/HF, SDNN, RMSSD, SDSD, NN50, pNN50, HTI, SD_1_, SD_2_
Veerabhadrappa et al [26], 2015, India	Cross-sectional study with 4 groups	156 participants; first trimester (n=25), second trimester (n=47), third trimester (n=52), and postpartum (within a week; n=32)	A single, simultaneous assessment in each group	ECG	LF (nu), HF (nu), LF/HF
Stein et al [27], 1999, United States	Longitudinal observational study with a single group	8 healthy nonpregnant individuals who expect to be pregnant	5 successive 24-hour recordings at prepregnancy and ≤6, 10, 18, and 34 weeks of gestational age	Holter	ULF^m^, VLF, LF (ms^2^), HF (ms^2^), SDNN, SDANN^n^, SDNN, RMSSD, pNN50, TP

^a^ECG: electrocardiogram.

^b^LF: low frequency.

^c^HF: high frequency.

^d^SDNN: standard deviation of the NN interval.

^e^RMSSD: root mean square of successive NN interval differences.

^f^pNN50: percentage of successive NN intervals that differ by more than 50 ms.

^g^HTI: integral of the intensity of the NN interval histogram divided by its height.

^h^TINN: total variation index of the NN intervals.

^i^VLF: very-low frequency.

^j^SDSD: standard deviation of the differences between successive NN intervals.

^k^SD_1_: Poincaré plot standard deviation perpendicular to the line of identity.

^l^SD_2_: Poincaré plot standard deviation along the line of identity.

^m^ULF: ultra-low frequency.

^n^SDANN: standard deviation of the average NN interval.

#### HRV Assessment

##### Tools

To measure HRV components, the majority of the studies (7/8, 88%) computed the short-term beat-to-beat interval using an electrocardiogram (ECG). Only 1 study used a 24-hour Holter for HRV assessment. These assessment tools, however, used different software to analyze the produced algorithm, such as VarioWin_HR (Genesis Medical Systems Pvt Ltd, Telangana, India), NI-DAQ approximate entropy (ApEn; National Instruments Corp, Austin, TX), DATAQ Instruments (Akron, OH), Labchart Pro 7 (ADInstruments, Sydney, Australia), and a Marquette Laser SXP Holter scanner (Marquette Electronics Inc, Milwaukee, WI). One study did not report the software used for signal analysis.

##### Assessment Characteristics

Assessment processes were performed in health settings such as a hospital or clinic. In 3 of 8 studies, participants were asked to refrain from consuming stimulant substances including tea, coffee, cola, and alcoholic drinks as well as to refrain from smoking for 24 hours before the testing [21,23,24]. In 2 of 8 studies, the time frame for conducting the ECG test was specified to be between 8 am and 9 am to 12 pm [21,24]. In 5 of 8 studies, the ECG test was acquired in the supine position [21-23,25]. In 4 of 8 studies, participants were asked to rest for 5 minutes [21,25], 10 minutes [24], or 20 minutes [23] before undergoing the ECG test. The duration of the ECG record varied in different studies and was either 5 minutes [21,23-25] or 10 minutes [20,22]. One study did not specify the duration [26]. Of the 8 studies, 3 used only lead II to obtain the ECG signals [23-25]. The rest of the studies did not report any information regarding the leads that were used.

##### Assessed Metrics

In the included studies, in general, HRV was operationalized using 3 types of components: time-domain (TD), frequency-domain (FD), and nonlinear methods. TD is the primary and simplest way to calculate HRV using statistical calculations of several consecutive beat-to-beat (RR) intervals, and a TD graph shows how a signal changes over time. FD represents a model that reflects the strength of the ANS function (specifically the parasympathetic branch) at a given time, and an FD graph shows how much of the signal lies within each given frequency band over a range of frequencies. The representative metrics for FD include the average NN interval, low frequency (LF), high frequency (HF), the LF/HF ratio, very-low frequency (VLF), ultra-low frequency (ULF), and total power (TP). The relevant metrics for TD are the standard deviation of the NN interval (SDNN), root mean square of successive NN interval differences (RMSSD), successive NN intervals that differ by more than 50 ms (NN50), percentage of NN50 (pNN50), standard deviation of the differences between successive NN intervals (SDSD), and integral of the intensity of the NN interval histogram divided by its height (HTI). A few studies also considered nonlinear algorithms such as the Poincaré plot standard deviation perpendicular to the line of identity (SD_1_) and Poincaré plot standard deviation along the line of identity (SD_2_) to measure HRV during pregnancy [11]. The detailed descriptions of all these HRV components including TD, FD, and nonlinear metrics are provided in the Table 2.

Of the 2 main HRV components (TD and FD), at least 3 indexes of FD metrics (eg, LF, HF, LF/HF, VLF, ULF, average NN [RR] interval, TP) were assessed in all the included studies. TD indexes, including SDNN, RMSSD, NN50, pNN50, SDSD, HTI, and TP, were also assessed. The nonlinear methods of SD_1_ and SD_2_ were acquired in 2 of 8 studies.

HRV changes or an HRV pattern was defined in the studies as an increase or decrease in the aforementioned HRV components. For example, SDRR is defined as standard deviation of the RR interval. If an article reported an increased SDRR from the first to the second trimesters, the SD of the RR interval was increased from the first trimester to the second trimester.

**Table 2:** Heart rate variability (HRV) components and metrics.

Components and metrics		Unit	Description
**Time domain**			
	SDNN	ms	Standard deviation of the NN interval
	RMSSD	ms	Root mean square of successive NN interval differences
	NN50	ms	Mean number of times an hour in which the change in successive normal sinus (NN) intervals exceeds 50 ms
	pNN50	%	Percentage of successive NN intervals that differ by more than 50 ms
	SDSD	ms	Standard deviation of the differences between successive NN intervals
	HTI	N/A^a^	Integral of the intensity of the NN interval histogram divided by its height.
**Frequency domain**			
	LF	ms^2^/nu	Absolute/relative power of the low frequency band (0.04-0.15 Hz)
	HF	ms^2^/nu	Absolute/relative power of the high frequency band (0.15-0.4 Hz)
	LF/HF	%	Ratio of LF to HF
	ULF	ms^2^	Absolute power of the ultra-low frequency band (≤0.003 Hz)
	VLF	ms^2^	Absolute power of very-low frequency band (0.0033-0.04 Hz)
	Avg N-N	ms	Mean of the NN intervals
	TP	ms^2^	Absolute power of the total frequency band (≤0.4 Hz)
**Nonlinear**			
	SD_1_	ms^2^	Poincaré plot standard deviation perpendicular to the line of identity
	SD_2_	ms^2^	Poincaré plot standard deviation along the line of identity

^a^N/A: not applicable.

### HRV Changes in Different Trimesters as Compared With Nonpregnant Individuals

The nonpregnant comparison group included individuals who did not report pregnancy at the time of assessment or were postpartum. Of the studies, 75% (6/8) reported HRV changes in pregnancy as compared with nonpregnancy. However, not all 6 studies overlapped in terms of the assessed HRV components. With this heterogeneity, the data for a trend assessment across the studies were insufficient for most of the HRV components. To report the frequency of the assessed metrics, we used the report format of “X out of Y increased/decreased” in which Y represents the total number of studies that reported the intended component and X reflects the number of studies in which X decreased or increased. The direction of change for most of the HRV components in both FD and TD indexes varied in different studies, specifically in early pregnancy. However, some FD and TD elements showed the same change direction (increase or decrease) in late pregnancy in the majority of the studies that reported the HRV. For instance, as compared with nonpregnant individual elements, the FD elements of HF (nu) in 75% (3/4) of the studies, HF (ms^2^) in 100% (3/3) of the studies, and VLF in 100% (3/3) of the studies decreased, and LF (nu) increased in late pregnancy in 75% (3/4) of the studies. The TD components of SDNN in 80% (4/5) of the studies, RMSSD in 100% (5/5) of the studies, and pNN50 in 80% (4/5) of the studies decreased in late pregnancy as compared with nonpregnant individuals. See Table 3 for more details.

**Table 3:** Changes in heart rate variability (HRV) components in pregnant individuals as compared with nonpregnant individuals in different trimesters.

First author, year, and measurement period			Linear: frequency domain (FD)																	Linear: time domain (TD)															Nonlinear		
			LF^a^		HF^b^		LF (nu)		HF (nu)		LF/HF		ULF^c^		VLF^d^		Average NN		TP^e^			SDNN^f^		RMSSD^g^		NN50^h^		pNN50^i^		SDSD^j^		HTI^k^		SD_1_^l^			SD_2_^m^
**Gandhi [25], 2014**																																					
	T_1_^n^	NC^o^		NC		NC		NC		NC		—^p^		NC		—		—			NC		NC		NC		NC		NC		—		NC			NC	
	T_3_^q^	D^r^		D		I^s^		D		D		—		D		—		—			D		D		D		D		D		—		I			I	
**Stein [27], 1999**																																					
	T_1_	D		D		—		—		—		D		D		—		D			D		D		—		D		—		—		—			—	
	T_3_	D		D		—		—		—		D		D		—		D			D		D		—		D		—		—		—			—	
**Chamchad [22], 2007**																																					
	T_3_	NC		NC		—		—		I		—		—		D		—			D		D		—		D		—		D		D			D	
**Solanki** **[21]** **, 2020**																																					
	T_1_	—		—		D		I		D		—		D		—		—			D		D		I		I		I		I		D			D	
	T_2_^t^	—		—		D		I		D		—		D		—		—			D		D		I		I		I		I		D			D	
	T_3_	—		—		D		I		D		—		D		—		—			D		D		I		I		I		I		D			D	
**Alam** **[24]** **, 2018**																																					
	T_1_	D		I		D		I		D		—		—		I		—			I		I		I		I		—		—		—			—	
	T_2_	I		D		I		D		I		—		—		D		—			I		I		D		D		—		—		—			—	
	T_3_	I		D		I		D		I		—		—		D		—			I		D		D		D		—		—		—			—	
**Veerabhadrappa** **[26]** **, 2015**																																					
	T_1_	—		—		I		D		I		—		—		—		—			—		—		—		—		—		—		—			—	
	T_2_	—		—		I		D		I		—		—		—		—			—		—		—		—		—		—		—			—	
	T_3_	—		—		I		D		I		—		—		—		—			—		—		—		—		—		—		—			—	

^a^LF: low frequency.

^b^HF: high frequency.

^c^ULF: ultra-low frequency.

^d^VLF: very-low frequency.

^e^TP: total power.

^f^SDNN: standard deviation of the NN interval.

^g^RMSSD: root mean square of successive NN interval differences.

^h^NN50: successive NN intervals that differ by more than 50 ms.

^i^pNN50: percentage of successive NN intervals that differ by more than 50 ms.

^j^SDSD: standard deviation of the differences between successive NN intervals.

^k^HTI: integral of the intensity of the NN interval histogram divided by its height.

^l^SD_1_: Poincaré plot standard deviation perpendicular to the line of identity.

^m^SD_2_: Poincaré plot standard deviation along the line of identity.

^n^T1: first trimester.

^o^NC: no change.

^p^Not applicable.

^q^T_3_: third trimester.

^r^D: decrease.

^s^I: increase.

^t^T_2_: second trimester.

### HRV Adaptation During Different Trimesters of Pregnancy

According to the literature, ANS regulation during pregnancy starts in the first weeks of pregnancy and continues until the end of pregnancy. These changes vary based on the pregnancy-related time-sensitive demands to ensure fetus development. To understand the potential differences in HRV components between different trimesters, for this literature review, HRV changes were assessed in transition between different trimesters during pregnancy**.**

#### First Trimester to the Third Trimester

HRV changes during the first and third trimesters of pregnancy were assessed in 7 (7/8, 88%) studies. In all 7 studies, whether they were longitudinal studies with the same population or cross-sectional studies with different populations in different trimesters, the findings for most of the FD and TD indexes were analogous. Most of the TD metrics generally decreased from the first trimester to the third trimester in the majority (5/7, 71%) of the studies. Of the TD indexes, the following decreased during pregnancy: SDNN in 80% (4/5) of the studies, RMSSD in 75% (3/4) of the studies, NN50 in 70% (2/3) of the studies, PNN50 in 80% (4/5) of the studies, SDSD in 70% (2/3) of the studies, and SD_1_ and SD_2_ in 50% (1/2) of the studies. The FD indexes also decreased most of the time except for normalized LF (nu) and the LF/HF ratio, which showed an ascending trend from early to late pregnancy. Among the various FD indexes, LF power (ms^2^) in 60% (3/5) of the studies, HF power (ms^2^) in 100% (5/5) of the studies, HF (nu) in 100% (5/5) of the studies, VLF in 75% (3/4) of the studies, TP in 100% (2/2) of the studies, and the average NN interval in 100% (2/2) of the studies decreased, while LF (nu) in 80% (4/5) of the studies and LF/HF in 100% (6/6) studies increased. See Table 4 for more details.

**Table 4:** Changes in heart rate variability (HRV) components during pregnancy from early (first trimester) to late pregnancy (third trimester).

First author, year	Linear frequency domain (FD)										Linear time domain (TD)							Nonlinear
	LF^a^	HF^b^	LF (nu)	HF (nu)	LF/HF	ULF^c^	VLF^d^	Average NN	TP^e^	SDNN^f^		RMSSD^g^	NN50^h^	pNN50^i^	SDSD^j^	HTI^k^	SD_1_^l^ and SD_2_^m^	
Puente [20], 2011	NC^n^	D^o^	—^p^	—	I^q^	—	I	—	—	—		—	—	—	—	—	—	
Garg [23], 2020	D	D	I	D	I	—	—	—	D	D		—	—	D	D	—	—	
Gandhi [25], 2014	D	D	I	D	I	—	D	D	—	D		D	—	D	D	D	D	
Stein [27], 1999	D	D	—	—	—	D	D	—	D	D		D	D	D	—	—	—	
Solanki [21], 2020	—	—	D	D	I	—	D	—	—	NC		NC	NC	NC	NC	NC	NC	
Alam [24], 2018	I	D	I	D	I	—	—	D	—	D		D	D	D	—	—	—	
Veerabhadrappa [26], 2015	—	—	I	D	I	—	—	—	—	—		—	—	—	—	—	—	

^a^LF: low frequency.

^b^HF: high frequency.

^c^ULF: ultra-low frequency.

^d^VLF: very-low frequency.

^e^TP: total power.

^f^SDNN: standard deviation of the NN interval.

^g^RMSSD: root mean square of successive NN interval differences.

^h^NN50: successive NN intervals that differ by more than 50 ms.

^i^pNN50: percentage of successive NN intervals that differ by more than 50 ms.

^j^SDSD: standard deviation of the differences between successive NN intervals.

^k^HTI: integral of the intensity of the NN interval histogram divided by its height.

^l^SD_1_: Poincaré plot standard deviation perpendicular to the line of identity.

^m^SD_2_: Poincaré plot standard deviation along the line of identity.

^n^NC: no change.

^o^D: decrease.

^p^Not applicable.

^q^I: increase.

#### First Trimester to the Second Trimester

Only one-half (4/8, 50%) of the studies reported HRV changes from the first trimester to the second trimester. The included articles either did not report TD and FD or were divergent in measured TD and FD indexes from the first to the second trimesters of pregnancy. Thus, determining a general pattern relying on the current findings cannot be done. However, the elements often tended to decline or stay unchanged from the first to the second trimesters except for normalized LF (nu) and LF/HF, which increased in 75% (3/4) and 100% (4/4), respectively, of the reported elements. See Table 5 for more details.

**Table 5:** Changes in heart rate variability (HRV) components from the first to the second trimesters of pregnancy.

First author, year	Linear frequency domain (FD)										Linear time domain (TD)							Non linear
	LF^a^	HF^b^	LF (nu)	HF (nu)	LF/HF	ULF^c^	VLF^d^	Average NN	TP^e^	SDNN^f^		RMSSD^g^	NN50^h^	pNN50^i^	SDSD^j^	HTI^k^	SD_1_^l^ and SD_2_^m^	
Garg et al [23], 2020	D^n^	D	I^o^	D	I	—^p^	—	—	D	D		—	—	D	D	—	—	
Solanki [21], 2020	—	—	D	D	I	—	D	—	—	NC^q^		NC	NC	NC	NC	NC	NC	
Alam et al [24], 2018	I	D	I	D	I	—	—	D	—	D		D	D	D	—	—	—	
Veerabhadrappa [26], 2015	—	—	I	D	I	—	—	—	—	—		—	—	—	—	—	—	

^a^LF: low frequency.

^b^HF: high frequency.

^c^ULF: ultra-low frequency.

^d^VLF: very-low frequency.

^e^TP: total power.

^f^SDNN: standard deviation of the NN interval.

^g^RMSSD: root mean square of successive NN interval differences.

^h^NN50: successive NN intervals that differ by more than 50 ms.

^i^pNN50: percentage of successive NN intervals that differ by more than 50 ms.

^j^SDSD: standard deviation of the differences between successive NN intervals.

^k^HTI: integral of the intensity of the NN interval histogram divided by its height.

^l^SD_1_: Poincaré plot standard deviation perpendicular to the line of identity.

^m^SD_2_: Poincaré plot standard deviation along the line of identity.

^n^D: decrease.

^o^I: increase.

^p^Not applicable.

^q^NC: no change.

#### Second Trimester to the Third Trimester

Only one-half (4/8, 50%) of the studies reported HRV changes from the second to the third trimesters. Divergency in the TD and FD metrics considered in studies did not allow for the comparative assessment between studies. Nevertheless, the changes mostly were similar to those from the first to the second trimesters except for normalized LF (nu), which was neutral; HF (nu), which decreased in 75% (3/4) of the studies; and the LF/HF ratio, which increased in 75% (3/4) of the studies that measured the HRV from the second trimester to the third trimester. See Table 6 for more details.

**Table 6:** Changes in heart rate variability (HRV) components from the second to the third trimesters of pregnancy.

First author, year	Linear frequency domain (FD)										Linear time domain (TD)							Nonlinear
	LF^a^	HF^b^	LF (nu)	HF (nu)	LF/HF	ULF^c^	VLF^d^	Average NN	TP^e^	SDNN^f^		RMSSD^g^	NN50^h^	pNN50^i^	SDSD^j^	HTI^k^	SD_1_^l^ and SD_2_^m^	
Garg et al [23], 2020	D^n^	D	D	I^o^	D	—^p^	—	—	D	D		—	—	D	D	—	—	
Solanki [21], 2020	—	—	D	D	I	—	D	—	—	NC^q^		NC	NC	NC	NC	NC	NC	
Alam et al [24], 2018	I	D	I	D	I	—	—	D	—	I		D	D	D	—	—	—	
Veerabhadrappa [26], 2015	—	—	I	D	I	—	—	—	—	—		—	—	—	—	—	—	

^a^LF: low frequency.

^b^HF: high frequency.

^c^ULF: ultra-low frequency.

^d^VLF: very-low frequency.

^e^TP: total power.

^f^SDNN: standard deviation of the NN interval.

^g^RMSSD: root mean square of successive NN interval differences.

^h^NN50: successive NN intervals that differ by more than 50 ms.

^i^pNN50: percentage of successive NN intervals that differ by more than 50 ms.

^j^SDSD: standard deviation of the differences between successive NN intervals.

^k^HTI: integral of the intensity of the NN interval histogram divided by its height.

^l^SD_1_: Poincaré plot standard deviation perpendicular to the line of identity.

^m^SD_2_: Poincaré plot standard deviation along the line of identity.

^n^D: decrease.

^o^I: increase.

^p^Not applicable.

^q^NC: no change.

### Risk of Bias in the Studies

**Figure 2:**
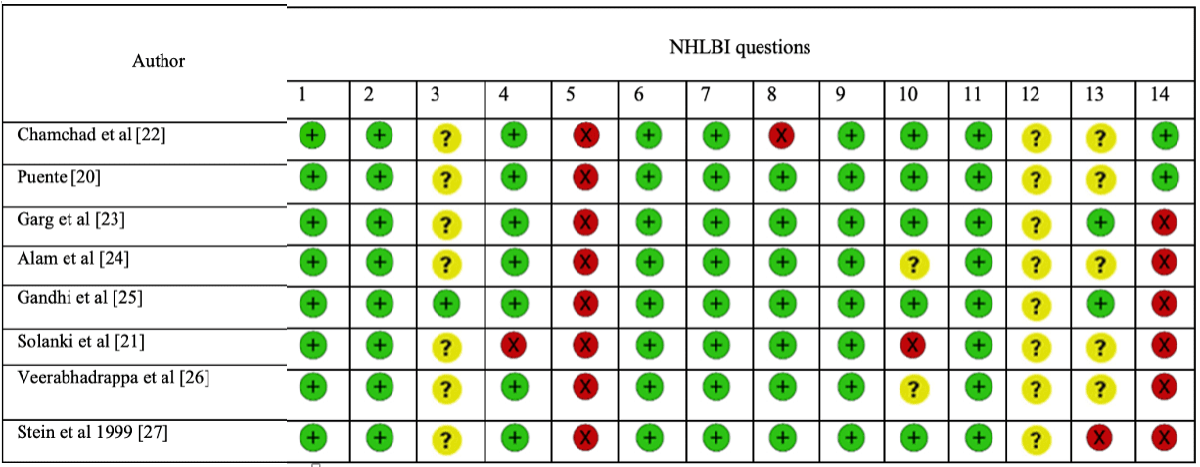
National Heart, Lung, and Blood Institute (NHLBI) quality assessment tool, with which the study quality was scored based on the number of addressed domains (≤7 poor; 8 fair; ≥9 good): (1) clear research objective; (2) clear study population; (3) participation rate >50%; (4) internal validity in the population; (5) sample size justification; (6) prospective study; (7) time span between exposure and outcome; (8) exposure aspects; (9) exposure measures; (10) exposure assessment frequency; (11) outcome aspects; (12) blinded outcome assessment; (13) attrition; (14) confounding factors.

The result of the NHLBI assessment is reported in Figure 2. Articles were peer-reviewed in Covidence online software by 2 independent reviewers. All the studies were screened by both reviewers, ZS and JO, in 2 steps. In the first step, the quality of articles was assessed using the 14 domains of the NHLBI see Multimedia Appendix 2 for more details). Next, the overall quality of each study was assessed based on the addressed NHLBI domains. To resolve raised disagreements, a third reviewer, MB, was involved. The research question, study population, dependent variables, and independent variables were specified in all the studies. Since the independent variable was “being pregnant,” it was already established in the study populations. The dependent variable was HRV, which may be affected by pregnancy. Both variables were consistent across the study populations in all the studies. The sample size was justified in none of the studies. In all the studies (8/8, 100%), the timeframe was sufficient for the potential expected association to occur; the study population was pregnant individuals who were already pregnant when the HRV assessment was performed. The overall quality of the studies was based on the number of domains addressed in each study.

## Discussion

### Principal Findings

In this study, we aimed to review studies concerned with HRV adaptation to evaluate ANS function in healthy pregnant individuals. According to our findings based on the existing data, during pregnancy, almost all the TD and most of the FD bands were decreased except for LF (nu) and the LF/HF ratio. From the second trimester to the third trimester, however, LF change was not consistent across the studies; for example, one-half of the studies showed a decrease, while the other one-half demonstrated an increase in LF. Increased LF/HF and LF during pregnancy indicate a dominance of the sympathetic nervous system over the parasympathetic nervous system. This result is in accordance with the findings in the existing literature, in which methods other than the HRV were used for ANS assessment. For example, Ekholm et al [28] used various maneuvers including the Valsalva maneuver, deep breathing test, orthostatic test, and isometric handgrip test to assess ANS changes in pregnancy. They concluded that the parasympathetic nervous system becomes less active as time progresses in pregnancy [28]. Kochhar et al [29] also applied conventional tests such as the standing-to-lying down ratio, Valsalva maneuver, tachycardia maneuver, hand grip test, and cold pressor test to assess the ANS in pregnancy. The results supported sympathetic activation over parasympathetic activation from the first through the third trimesters and compared with nonpregnant women [29]. Systematic reviews have also investigated the application of HRV for ANS assessment in complicated pregnancies and showed that HRV as a biomarker of the ANS can be affected by common pregnancy complications including hypertensive disorders such as preeclampsia [28,29]. These studies suggested that parasympathetic activity of the ANS decreases more than sympathetic activity in hypertensive pregnant women. This may explain why hypertension often occurs in the late second or third trimester of pregnancy when the physiological response tends to be sympathetic overactivation.

### Synthesis of the Results and Limitations

The heterogeneity in the findings from the included studies can potentially be explained by the methodological issues we uncovered, which threaten the internal and external validity of the findings. Due to the divergence in the selected and reported HRV components by different studies, a reliable conclusion regarding ANS function cannot be reached using these insufficient data. Additionally, the wide variability in the length of the recording period may have significantly affected both FD and TD measurements [30]. A short-term epoch (~5 minutes) lacks the prognostic potential for morbidity and mortality. Basically, in published protocols, the recommended assessment periods for HRV recordings vary from 1 minute to 24 hours for various FD and TD metrics [11]. However, since important factors including circadian rhythms, metabolism, the sleep cycle, core body temperature, and the renin-angiotensin system follow a 24-hour cycle, the length of clinical HRV assessments should be at least 24 hours to provide acceptable information [11]. The studies that were reviewed in this study often used 5-minute to 10-minute assessments, which may make it challenging to achieve reliable results. Furthermore, the HRV measurement in the included studies was often conducted in clinic or hospital settings, and the studies often ignored the impact of mental-environmental confounding factors including negative mental situations such as stress, anxiety, and fear and environmental factors (eg, temperature) that can temporarily affect ANS function, potentially leading to false results. These factors could be significantly associated with ANS function and thus may lead to variability in and misinterpretation of ANS function in response to pregnancy. Also, the frequency of HRV measurements varied from 1 to 3 times a trimester in different studies. An episodic assessment (ie, 1 or 3 times a trimester) may not be reflective of actual ANS function in real life in response to the ever-changing pregnancy-related demands. This is because the ANS is a responsive system that continuously undergoes dynamic adaptations in response to the various internal and external situations that one may face from moment to moment [8].

Another inconsistency we found in the included studies was the way they operationalized HRV. As discussed earlier, HRV was operationalized in various ways, via TD, FD, and nonlinear methods, each involving different corresponding components. No justification was provided in any study for the selection of HRV measurement modality and associated components. Specifying the weaknesses and strengths of the applied components for measurement may provide important information for future studies. It is critical to address why some components are commonly used as compared with others to represent HRV and whether the applied component is reliable, valid, and easily measured. For example, recent studies showed that linear algorithms including TD and FD are affected by nonstationarity and thus perhaps not adequate for HRV assessment [31]. Nonlinear (fractal) measurements such as SD_1_, SD_2_, ApEn, and sample entropy are recommended as they represent the unpredictability of a time series resulting from the complexity of the regulatory mechanisms of HRV. It is suggested that nonlinear HRV measures may enable clinicians and researchers to study the complex interactions between electrophysiological, hemodynamic, and humoral variables as well as their regulation by the ANS and central nervous system [31].

One of the limitations of this study is that its protocol was not registered in PROSPERO.

### Implications

This study will help us determine if there are consistent stable patterns of HRV across pregnancy for a sample of healthy pregnant women that reflect “healthy” ANS function. Determination of the pattern can provide the basis for extended research in order to determine if the identified pattern is generalizable to a larger sample of pregnant individuals and then in other, diverse pregnant populations. If a pattern is found, then the next step would be to compare the pattern with complicated pregnancies to find where, when, and how this pattern may be different. Understanding the differences between complicated and healthy pregnancies can provide an opportunity to develop a screening and detective system to screen all pregnant women throughout pregnancy and predict whether they are manifesting the nonhealthy pattern, to be able to perform a timely intervention before it threatens the mother’s and baby’s lives. Due to the limitations in the existing literature, identifying potential patterns seems critical. Thus, more studies are needed to reflect this pattern by eliminating the methodological limitations in current studies.

### Conclusion

This study determined the feasibility of HRV measurements to assess ANS function in pregnant individuals. We found significant heterogeneity in the HRV measurement modalities used, the settings in which measurements were performed, the time frames of the HRV assessment, and assessments done across trimesters. There were inconsistencies in definitions of healthy pregnancy across studies. We found some potential HRV patterns that were consistent within and across studies, but not all the studies were convergent in terms of the reported results, which may be due to the methodological heterogeneity. In summary, we found significant variability in how studies measured HRV and how they identified HRV patterns, which made it impossible to determine potentially normal HRV patterns across trimesters with any degree of validity. More research is needed to overcome the aforementioned limitations and determine the required criteria and methods to assess HRV patterns corresponding to ANS function in healthy pregnant persons.

## Appendix

Multimedia Appendix 1 Search strategy.

Multimedia Appendix 2 Quality assessment domains of the National Heart, Lung, and Blood Institute (NHLBI).

## Abbreviations

ANS: autonomic nervous system
ApEn: approximate entropy
ECG: electrocardiogram
HF: high frequency
HRV: heart rate variability
HTI: integral of the intensity of the NN interval histogram divided by its height
LF: low frequency
NHLBI: National Heart, Lung, and Blood Institute
NN50: successive NN intervals that differ by more than 50 ms
PECO: population, exposure, comparator, and outcome
pNN50: percentage of NN50 (pNN50)
PRISMA: Preferred Reporting Items for Systematic Reviews and Meta-Analyses
RMSSD: root mean square of successive NN interval differences
SD1: Poincaré plot standard deviation perpendicular to the line of identity
SD2: Poincaré plot standard deviation along the line of identity
SDSD: standard deviation of the differences between successive NN intervals
SDNN: standard deviation of the NN interval
TP: total power
ULF: ultra-low frequency
VLF: very-low frequency
