# Supplementary material for: The Utilization of Heart Rate Variability for Autonomic Nervous System Assessment in Healthy Pregnant Women: Systematic Review
Source: JMIR Bioinform Biotechnol. 2022 Nov 17;3(1):e36791. doi: 10.2196/36791 (PMC11135217; doi:10.2196/36791)
Supplement: Multimedia Appendix 1 [file bioinform_v3i1e36791_app1.docx]

Table S1. Search strategy.

| **Databases** | **Participants** | **Variables** | **Time span** | **Inclusion** | **Exclusion** |
| --- | --- | --- | --- | --- | --- |
|  | **Pregnant individuals** | **Heart rate variability** |  |  |  |
| PubMed | ("Pregnancy"[Majr:NoExp] | "heart rate variability"[ti] | 1996-June 2021 | English language | Systematic review, protocol, conference, letter to the editor, unpublished/under review papers, dissertation proposal. Book chapters |
|  | OR |  |  |  |  |
|  | pregnan*[ti]) |  |  |  |  |
| CINAHL | ((MM "Pregnancy") | ((MM "Heart Rate Variability") | 1961-June 2021 |  |  |
|  | OR | OR |  |  |  |
|  | TI pregnan*) | TI "Heart Rate Variability") |  |  |  |
| SCOPUS | TITLE (pregnan*)) | (TITLE ("Heart Rate Variability”) | 1970-June 2021 |  |  |
| Web of Science | "Heart Rate Variability" (Title)) | pregnan* (Title | 1900-June 2020 |  |  |
| AND, OR = Boolean operators  [Majr] = the topic is one of the main focuses of the article. [Majr:NoExp]  = the topic is one of the main focuses of the article, AND more specific terms NOT included: [ti] = term searched in the title of the article only  (MH "Pregnancy+") = more specific terms WEERE included (see below). MM = the topic is one of the main focuses of the article. [ti] = term searched in the title of the article only. (MH "Pregnancy Complications") NOT included.  TITLE-ABS-KEY = Term searched in the article title, abstract, and author-supplied keywords. TITLE = Term searched in the article title only | | | | | |
