# Supplementary figures and images for: The Utilization of Heart Rate Variability for Autonomic Nervous System Assessment in Healthy Pregnant Women: Systematic Review
Source: JMIR Bioinform Biotechnol. 2022 Nov 17;3(1):e36791. doi: 10.2196/36791 (PMC11135217; doi:10.2196/36791)

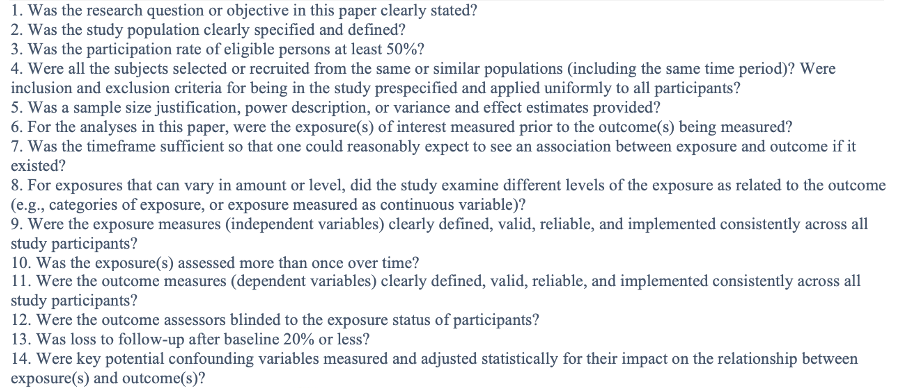

Supplement: Multimedia Appendix 2 [file bioinform_v3i1e36791_app2.png]
